# Barriers and facilitators to implementing immersive virtual reality in long-term care settings: an interdisciplinary partnership study exploring staff perspectives

**DOI:** 10.3389/fpain.2026.1734386

**Published:** 2026-01-29

**Authors:** Isabel Sadowski, Mael Gagnon-Mailhot, Gbeada Josiane Seu, Charles Sebiyo Batcho, Maude Laberge, Bassam Khoury, Gabriel Tremblay, Olivier Dubé, Charles Goyette, Antoine Rheault, Stephanie Glegg, Josiane Bissonnette, Carol Hudon, Anouk Lamontagne, Guillaume Léonard, Martin Lepage, Serge Marchand, Pierre Rainville, Alexandra Ribon-Demars, Harmehr Sekhon, Diane Tapp, Élisabeth Thibaudeau, Martine Bordeleau

**Affiliations:** 1 Department of Educational and Counselling Psychology, McGill University, Montreal, QC, Canada; 2 Department of Psychology, Université de Montréal, Montreal, QC, Canada; 3 Research Centre of the Centre Hospitalier de l’Université de Montréal, Montreal, QC, Canada; 4 Faculty of Nursing, Université Laval, Quebec City, QC, Canada; 5 VITAM—Centre de recherche en santé durable, Quebec City, QC, Canada; 6 Centre for Interdisciplinary Research in Rehabilitation and Social Integration (Cirris), CIUSSS de la Capitale-Nationale, Quebec City, QC, Canada; 7 School of Rehabilitation Sciences, Faculty of Medicine, Université Laval, Quebec City, QC, Canada; 8 Department of Social and Preventative Medicine, Faculty of Medicine, Université Laval, Quebec City, QC, Canada; 9 Centre de recherche du CHU de Québec, Université Laval, Quebec City, QC, Canada; 10 Department of Occupational Science & Occupational Therapy, Faculty of Medicine, University of British Columbia, Vancouver, BC, Canada; 11 Faculty of Music, Université Laval, Quebec City, QC, Canada; 12 Centre de recherche OICRM, Université Laval, Quebec City, QC, Canada; 13 School of Psychology, Université Laval, Quebec City, QC, Canada; 14 School of Physical and Occupational Therapy, McGill University, Montreal, QC, Canada; 15 Feil and Oberfeld Research Centre, Jewish Rehabilitation Hospital Site of CRIR and CISSS de Laval, Laval, QC, Canada; 16 Research Centre on Aging, CIUSSS de l’Estrie-CHUS, Sherbrooke, QC, Canada; 17 Faculty of Medicine and Health Sciences, Université de Sherbrooke, Sherbrooke, QC, Canada; 18 Department of Psychiatry, McGill University, Montreal, QC, Canada; 19 Department of Psychology, McGill University, Montreal, QC, Canada; 20 Douglas Research Centre, Douglas Mental Health University Institute, Montreal, QC, Canada; 21 Alan Edwards Centre for Research on Pain, McGill University, Montreal, QC, Canada; 22 Faculty of Dental Medicine, Université de Montréal, Montreal, QC, Canada; 23 Division of Geriatric Medicine and Department of Psychiatry, McGill University, Montreal, QC, Canada; 24 St. Mary’s Research Centre, Saint Mary’s Hospital, Montreal, QC, Canada; 25 CERVO Brain Research Centre, Quebec City, QC, Canada

**Keywords:** barriers & facilitative factors, implementation, long-term care, older adults, staff perspectives, virtual reality

## Abstract

**Introduction:**

Immersive virtual reality (VR) has attracted growing interest in long-term care (LTC) as a potential tool to enhance well-being and alleviate pain. However, its effective implementation by LTC staff remains understudied. In a participatory action, mixed-methods study co-developed with knowledge users (LTC frontline staff and managers, and a VR developer), we applied the Decomposed Theory of Planned Behaviour (DTPB) to examine VR adoption in LTC and clarify barriers and facilitators to sustainable implementation.

**Methods:**

Knowledge users were consulted to design and develop a single-phase, cross-sectional, convergent mixed-methods study. LTC staff (*n* = 16) were then recruited to complete an online self-report questionnaire specific to staff adoption of VR through the assessment of attitudes, social norms, perceived behavioural control and facilitating conditions. Quantitative data were analyzed descriptively. Qualitative data underwent content analysis via DTPB-guided deductive and inductive codes.

**Results:**

LTC staff indicated overall favourable attitudes towards VR use, yet only 25% (*n* = 4) of participants rated VR as easy to use and 50% (*n* = 8) deemed it suitable for LTC settings. Staff confidence with VR use was moderate, with resident selection and troubleshooting highlighted as particular challenges. Barriers such as lacking time to learn and use VR systems, as well as potential resident discomfort, were highlighted. Facilitators included adequate activity space, organizational support, and person-centred delivery, which staff linked to residents’ relaxation, positive affect, and progressive engagement. Content analysis identified four key themes explaining these patterns: (1) Barriers outweighing promise, (2) Personalization of the VR experience, (3) Enabling conditions—Environment and organization, and (4) Staff ambivalence to VR.

**Discussion:**

VR adoption appears to be linked to alignment between compatibility, ease-of-use, facilitating conditions, and social support. Person-centred delivery and organizational support may enable consistent use and enhance residents’ relaxation, positive affect and progressive engagement, relevant to pain care. Findings offer a practical roadmap for integrating VR as a low-burden intervention, while highlighting areas needing further attention to promote sustainable implementation.

## 1 Introduction

By 2070, the Canadian population aged 65 and older, which was estimated at 7 million in 2022, is expected to double to between 14 and 16 million (1, 2). Concurrently, an estimated 14% of older Canadians report having a diagnosed mental health condition, with another 18% reporting emotional distress that was difficult to cope with during the past year (3). These rates increase to 25% to 50% for those residing in long-term care (LTC) facilities (4, 5). Emotional distress and depressive symptoms are therefore relatively commonplace in Canadian LTC settings, and closely tied to poorer quality-of-life, greater pain sensitivity, and chronic pain (6, 7). Chronic pain, in turn, is associated with social isolation (8), depression, anxiety (9, 10), reduced quality-of-life (11), and worsened functional decline (12). These figures underscore the need for accessible, effective, person-centred approaches to better manage older adults’ mental health and pain.

Immersive virtual reality (VR) technology has the potential to significantly improve the mental health and quality-of-life of older individuals receiving care at home or in institutions. This is due, in part, to advancements in VR technology (13, 14). Immersive VR technology provides a unique advantage by being able to simultaneously address the physical, psychological, and social needs of users (15, 16). VR can enhance emotional experience by enabling the discovery of new environments (17), while also providing distraction that can divert attention from pain (18–20) and negative emotions (21, 22), offering users a mental reprieve. Emerging evidence suggests that immersive and semi-immersive VR interventions are not only feasible but also well accepted by older adults with moderate to advanced cognitive impairment. For example, systematic reviews and feasibility trials report that individuals with dementia and mild cognitive impairment generally enjoy VR experiences, show improvements in mood and engagement, and tolerate the technology with minimal adverse effects (16, 23–25).

In addition, incorporating 360-degree nature videos into VR programs has been proposed as a strategy to enhance older adults' mental health and well-being (26–28). Immersive 360-degree VR programs offer a practical and low-cost solution to provide access to nature (27, 29). Studies suggest that 360-degree nature videos are effective for reducing pain and pain-related symptoms, physiological stress, and improving cognitive functioning and mood (30–33).

Beyond access to nature, immersive VR offers an engaging environment for practicing mindfulness, with potential applications in the context of aging (34–38). Mindfulness, defined as intentional present-moment awareness and acceptance, is theorized to be a key component of VR interventions (39–41). Mindfulness-based interventions have demonstrated positive effects on pain, stress, depression, anxiety, rumination, and sleep in older adults (42–48). Delivering mindfulness training through VR may be an optimal way to engage older adults (49, 50). Indeed, mindfulness-based programs using VR have been linked to improvements in chronic pain management through the technology's capacity to augment mindfulness practice (51). While both VR-based mindfulness and nature appear to have the potential to enhance older adults' overall well-being, few studies have assessed their feasibility, acceptability, or efficacy in LTC settings (52). Furthermore, there is limited research examining the factors contributing to optimal delivery of VR interventions from the perspective of staff working in LTC (53, 54).

In this study's context, “staff” refers specifically to frontline professionals in LTC homes who are tasked with delivering recreational and well-being programs to residents. These include recreation practitioners (e.g., recreation therapists, recreation technicians, recreation managers), specialized educators (psycho-educators and psychosocial workers), kinesiologists, physiotherapists, occupational therapists, and LTC care attendants. These staff members are often directly responsible for key aspects of intervention delivery, including technology set-up and facilitation of appropriate, supportive interactions with older adult residents (54). Given the crucial role that LTC staff play in the adoption and implementation of VR-related interventions (53, 54), there is a clear need to build understanding of factors that may influence staff motivation or well-being with respect to VR facilitation (e.g., organizational support, appropriate training and resources, self-efficacy with new technology, etc.) (28, 54–57). In a qualitative interview study, Wong and colleagues (2024) examined barriers and facilitators to VR use in the context of a research team-delivered LTC-based VR program. They found that when LTC staff viewed VR programs as acceptable, easy to use, and beneficial for residents, they were more likely to adopt and integrate VR into permanent service delivery. However, beyond qualitative mapping of researcher-delivered programs, there is a need for theory-driven studies of staff-led programs that both quantify technology adoption determinants (e.g., ease of use, facilitating conditions, self-efficacy) and expand upon these findings using qualitative methodology. Research in this area will help to clarify common trends in barriers and facilitators, as well as to highlight divergent, context-specific factors worth considering when tailoring VR programs for pain and mood management in LTC.

To address the current gaps in the literature, and better meet the needs of LTC residents and staff, our research team paired with the Montreal-based VR enterprise, *Super Splendide*. Since 2020, *Super Splendide* has been developing “Toujours Dimanche”, a multi-user VR application designed specifically for immersive VR headsets. At the time of the current study, the application offered 360-degree videos of natural environments and heritage sites, along with audio-guided mindfulness meditations linked to the VR videos. Previous work using the Toujours Dimanche application focused on community-dwelling older adults, where a VR nature-meditation program showed promising preliminary outcomes (58). Building on these findings, it is essential to explore whether similar benefits extend to LTC settings, where residents face unique challenges and staff play a central role in VR program implementation. Accordingly, a formal evaluation of staff perceptions of Toujours Dimanche's acceptability, alongside the broader acceptability of using VR with head-mounted displays, represents a crucial step towards effective development and implementation in the LTC context. This groundwork enables potential integration of VR-based programs for mood and pain management in LTC, where chronic pain and mental health conditions are highly prevalent and often under-managed despite their profound impact on quality-of-life and caregiving demands.

In this context, our study aimed to identify staff-perceived barriers and facilitators to adopting both VR and Toujours Dimanche in LTC, using a single-phase, cross-sectional, convergent mixed-methods survey. This research intended to build understanding of LTC staff's field experience with VR and Toujours Dimanche, and associated training connected to its use. It is part of a larger program of research examining the design and implementation of VR programs for older adults in LTC homes in Quebec, Canada.

## 2 Materials and methods

### 2.1 Research team

Following a participatory action research approach (59), our research team involved (*n* = 28 total) key stakeholders including three LTC staff knowledge users, two LTC organizational manager partners, one industry partner, two patient partners living in LTC, two family partners with caregiving experience for their family members residing in LTC, 14 researchers and/or clinicians, and four trainees. Throughout the research process, team meetings were held monthly to integrate the perspectives and suggestions of the stakeholders described above to improve the study's methodology and materials and to inform our objectives.

### 2.2 Study context

This study was part of a larger research program aiming to understand the best ways to effectively implement VR technology with older adults in LTC, as well as to explore the specific feasibility, acceptability, and efficacy of a multi-user VR app (i.e., Toujours Dimanche) in this context. Since 2020, *Super Splendide* has been developing the Toujours Dimanche VR application, which is also available on mobile devices (Android, iOS) to support VR program delivery by care practitioners. This study examined the use of Version 3 of Toujours Dimanche, which was released in 2023 and was a preliminary version of the application. *Super Splendide* has since been incorporating significant updates to the application for use in rural and urban LTC homes, run by provincial health organizations in Quebec, Canada. The company released Version 4 of their app in 2024. Version 3 of the Toujours Dimanche platform enabled staff to conduct VR sessions with an unlimited number of virtual users, immersing them in 360-degree videos of natural environments from the province of Quebec. Immersions involving audio-recorded, guided mindfulness meditations linked to the VR videos were also available to users. The application was developed to comply with provincial health and cybersecurity standards (60) and provided facilitators with administrative controls over in-headset content and settings. To date, the Toujours Dimanche application has been deployed in approximately 100 healthcare facilities, primarily in LTC and palliative care settings.

Furthermore, in 2022 the Chaudière-Appalaches public health authority [CISSS de Chaudière-Appalaches (CISSS-CA)] purchased approximately two Meta Quest 2 VR headsets per the 29 LTC homes in their region (61). *Super Splendide* was hired by the CISSS-CA in 2022 to provide VR training and support for LTC staff through in-person training workshops and an online community of practice. This was the company's first experience deploying services, training, and a community of practice with LTC organizations (during a period spanning from 2022 to 2023), and occurred in a context where approximately 75% of residents have neurocognitive disorders (62).

### 2.3 Participants, recruitment, and study sites

Our study focused on allied health professionals (e.g., recreation therapists, psychoeducators, physiotherapists), referred to as LTC staff, who work with people living in LTC residences based in the Chaudière-Appalaches region of Quebec, Canada, operated by the CISSS-CA. The residents of LTC homes in Quebec typically consist of older adults too frail to live on their own, who require 24/7 support [MSSS, 62]. The LTC staff targeted for this study work with residents presenting with conditions such as chronic pain, mobility issues, and significant cognitive challenges. They are also responsible for organizing VR-related activities with residents. LTC staff members were recruited for the study using convenience sampling. To be considered eligible, staff members were required to have completed the first version of the VR training delivered by *Super Splendide* during the 2022–2023 period, and to have used VR with LTC residents. Across the participating sites, approximately 28 LTC staff members met these eligibility criteria.

Initial recruitment was conducted using a network-based (informal referral) approach. Organizational partners in LTC on our research team (e.g., clinical activities specialists) promoted our study to staff via recruitment emails and posters. Recruitment was also conducted through in-person visits by the research team to LTC residences. Research team members then met with interested staff in-person or over the phone to answer their questions about the study.

### 2.4 Instruments

A sociodemographic questionnaire developed by the research team was used to collect information on participants (see Supplementary Appendix 1 for the sociodemographic questionnaire). The Assessing Determinants of Prospective Take-Up of Virtual Reality questionnaire [ADOPT-VR; 63; 64] assesses health professionals' intentions to use VR and perceived barriers/facilitators. The ADOPT-VR questionnaire has demonstrated strong face and content validity and good internal consistency (Cronbach's *α* = 0.876) (63). ADOPT-VR was used as a basis for the development of our mixed-methods French-language instrument (i.e., the “VR-use questionnaire”) for LTC staff. The specifically tailored VR-use questionnaire that was used for this study has not yet been psychometrically validated. It combined trichotomous items (yes/no/unsure), Likert-style confidence ratings (1-very uncomfortable; 5-very comfortable), and open-ended questions to assess individual, social, environmental, technology-specific, and system-level factors that influence LTC staff's adoption of VR over time.

Our VR-use questionnaire was field-tested to ensure content validity by asking the knowledge users and organizational partners on our research team for feedback on the clarity of wording, relevance of content, perceived gaps in content, repetitive questions, and general comments prior to being sent to participants. The developer of the original ADOPT-VR questionnaire (Dr. Stephanie Glegg) also provided feedback on the VR-use questionnaire during its development period. Our final questionnaire consisted of 67 items, using 34 trichotomous format items, five 5-point Likert-style items, two 0%–100% confidence scale items and 26 qualitative questions (see Supplementary Appendix 2 for the VR-use questionnaire).

### 2.5 Procedure

After eligible participants were contacted by the research team to answer their study-related questions, an email was sent to potential participants with a link to the consent form, which they completed through REDCap electronic data capture tools hosted at the Université de Sherbrooke (65). Participants who provided their written, informed consent were then able to access the study questionnaire, which they completed via REDCap. Participants received an email with the anonymous survey link. The link to the study survey was also available as a URL included at the end of the consent form on REDCap. The participants completed the demographics questionnaire and the mixed-methods VR-use questionnaire concurrently. The questionnaire was sent to participants over a period of eight months from July 2024 to February 2025.

### 2.6 Ethics

The program of research was reviewed and received ethics approval from the Chaudière-Appalaches Integrated Health and Social Services Centre (CISSS-CA) research ethics board, overseen by Quebec's provincial ministry of health (REB #: MP-23-2024-1086). Study participants provided their written informed consent to participate.

### 2.7 Study framework

The Decomposed Theory of Planned Behaviour [DTPB; 66], an extension of Ajzen's (67) Theory of Planned Behaviour (TPB) combined with the Technology Acceptance Model (68), explains technology adoption through deconstructing the three predictors of the TPB (i.e., attitudes, social norms, and perceived behavioural control) into actionable belief sets (e.g., perceived usefulness, ease of use, and compatibility for attitude; peer and supervisor influence for social norms; self-efficacy for perceived behavioural control). These constructs are modeled to predict behavioural intention and use, providing a structured lens to examine determinants of technology implementation. The DTPB framework guided the creation of the ADOPT-VR2 (63, 64) instrument, which was used to inform the development of our mixed-methods VR-use questionnaire. Our quantitative and qualitative findings were thus guided by the DTPB's framework, to enhance our analysis of the factors impacting LTC staff's adoption of VR. The modified VR-use questionnaire's constructs were grouped into the following composites, based on the DTPB: (1) Attitude: general attitude, perceived usefulness, perceived ease of use, compatibility; (2) Social Norms: client influence, peer influence, supervisor influence; (3) Perceived Behavioural Control: self-efficacy, behavioural intention; and (4) Facilitating Conditions: facilitators and barriers.

### 2.8 Data analysis

The data analysis followed a convergent mixed-methods approach, and integrated findings based on quantitative and qualitative consensus (69). Reporting followed Good Reporting of A Mixed Methods Study (GRAMMS) guidelines (70). Quantitative responses were analyzed using Microsoft Excel. Descriptive statistics were computed for all sociodemographic variables and the quantitative items of the VR-use questionnaire to summarize the data, explore associations, and identify key trends, using frequency counts, means or medians, and graphs, as appropriate. Quantitative results were grouped according to participant characteristics, DTPB domains, and findings specific to VR training quality.

Qualitative reporting followed the Standards for Reporting Qualitative Research (SRQR) (71). The responses to the qualitative questions were analyzed using content-analysis (72, 73), with deductive and inductive coding used to inform the analysis. Guided by the DTPB (66), we structured our analysis using deductive codes derived from its theoretical constructs, which were prepared pre-analysis in a clearly defined codebook, and confirmed for study alignment through team discussions.

Two master's level research trainees (GT and OD), supervised by PhD graduate students trained in qualitative analysis (MGM and IS), then conducted the preliminary data analysis. They started by repeatedly reading through the questionnaire responses to familiarize themselves with the data. Next, they identified meaning units (i.e., parts of the text relating to the research's aim), labeling each meaning unit with a code to briefly describe its content. Codes were either deductive (based on pre-analysis DTPB theoretical constructs), or inductive (developed from the data during the analysis). A coding list was used to clearly define codes and ensure consistency, and to note any changes in codes or evolutions that occurred. To improve reliability, the two research trainees independently coded the same portion of the data (equivalent to approximately 10% of the full dataset). To ensure intercoder reliability, coding decisions were then compared, discrepancies were discussed, and definitions were revised as needed with four additional members of the research team (IS, MGM, MB, CSB). After initial intercoder reliability review and adjustments, the remaining data was split evenly between the two trainees (GT, OD) to be coded. Additional reliability meetings with GT, OD, IS, MGM, MB and CSB were held after coding 30% and 70% of the data.

The coded meaning units were then checked by GT, OD, and IS with the full text to see if any relevant content was missed. These coding modifications were then reviewed and confirmed during a larger team meeting (GT, OD, IS, MGM, MB, CSB). Next, categorization of meaning units was conducted. GT and OD then identified codes describing similar ideas, which were grouped into subcategories. Subcategories were then grouped into broader categories which helped structure findings and were reviewed for accuracy and clarity by IS, MGM, MB and CSB. Next, GT and OD identified themes, which IS, MB, and MGM then confirmed. This process involved reflecting on the categories and developing higher-order concepts that captured underlying meanings (i.e., linking multiple categories at a more interpretive level); results were compiled in a table summarizing illustrative quotes, subcategories, categories, and themes.

Upon completion of both the quantitative and qualitative analyses, a structured, mixed-methods data integration meeting was held with seven research team members: IS, MGM, MB, CSB, GT, OD and CG. This meeting followed a systematic approach to compare whether findings confirmed, expanded, or diverged from each other, thereby enhancing both the meaning and reliability of the overall integrative interpretation (69). Mixed-methods results were synthesized and displayed using tables and illustrative quotations. Given that the questionnaires were completed in French, the quotes presented in this article have been translated into English and verified by bilingual team members.

Malterud and colleague's (74) information power principle guided this study's sample size determination. Prior to recruitment, a target of 16 participants was set based on this principle, which accounts for study aim, sample specificity, theoretical framework, and analytic strategy. This decision was informed by the limited literature on VR adoption in LTC, the theory-driven nature of our approach, and the specificity of our population (LTC staff trained in VR). Recruitment used convenience sampling from a defined pool of LTC staff who had completed VR training and used VR with residents. Organizational partners and the research team promoted the study via emails and posters, and the research team conducted in-person visits to LTC homes to answer questions and invite staff to participate in the study. These steps ensured that participants met eligibility criteria and reflected the intended population. A sample size of 16 participants falls within recommended ranges for qualitative descriptive studies (75–78) and provided sufficient information power for our mixed-methods design. Furthermore, within the specific setting from where we were recruiting, there were 28 eligible LTC staff. A sample size of 16 therefore represents 57.1% of the eligible population, which aligns with best-practice benchmarks for survey research (79, 80).

## 3 Results

### 3.1 Participant characteristics

Of the 20 people initially contacted to participate in the study, 17 provided consent and 16 completed the study questionnaires. The 16 retained participants had a mean age of 40.38 (*SD* = 8.07) and were white (100%; *n* = 16), francophone (100%; *n* = 16), and mostly women (81.3%; *n* = 13). Participants reported an average of 7.66 years of work experience across CHSLD, MDA, and MDAA settings. LTC staff members primarily identified as recreation practitioners (56.3%; *n* = 9) and specialized educators (31.3%; *n* = 5). Specialized educators in this context are psycho-educators and psychosocial workers who support residents' emotional and behavioral well-being and often facilitate individualized activities. For a more detailed overview of participant characteristics, see Table 1.

**Table 1:** Sociodemographic characteristics of study participants (*n* = 16).

Participant characteristics	Means (SDs), sample size and frequencies (%)
Age, *Mean* (*SD*), Range	40.38 (8.07), 26–58
Sex, *n* (%)	
Male	3 (18.75%)
Female	13 (81.25%)
Gender, *n* (%)	
Men	3 (18.75%)
Women	13 (81.25%)
Ethnicity, *n* (%)	
White	16 (100.00%)
First Language, *n* (%)	
French	16 (100.00%)
Additional Languages, *n* (%)	
English	4 (25.00%)
Years of Experience (LTC), *Mean* (*SD*), Range	7.66 (7.25), 26–50
LTC Role, *n* (%)	
Recreation Practitioners	9 (56.25%)
Specialized Educators	5 (31.25%)
Kinesiologist	1 (6.25%)
Other (LTC Attendant)	1 (6.25%)

Recreation Practitioners include recreation therapists, recreation technicians, recreation managers, and recreation educators; Specialized Educators include psycho-educators and psychosocial workers.

Regarding participants' prior VR training and experience, most participants (75.0%, *n* = 12) indicated that they had completed the one-day, 7-hour training offered by *Super Splendide* in 2022 and 2023, with others indicating that they had participated in the online training webinar offered by *Super Splendide* in 2023 (25.0%, *n* = 4). Two staff members (12.5%) reported participating in both the in-person and online training. Additionally, 3 participants (18.8%) indicated receiving supplementary online training delivered via video capsules, as part of *Super Splendide*'s online community of practice. With respect to VR use with LTC residents, staff reported an average of 1.5 VR sessions per month, with a mean usage time of approximately 17.6 min per session. Furthermore, on average, LTC staff reported delivering VR sessions to approximately 3 residents at a time. For more details, see Table 2.

**Table 2:** Characteristics of participants’ virtual reality training and usage (*n* = 16).

Participant responses	Response frequencies (*n* and %)
Completed 7 h Training	
Yes, *n* (%)	12 (75.00%)
No, *n* (%)	2 (12.50%)
No response, *n* (%)	2 (12.50%)
Completed Online Training Webinar	
Yes, *n* (%)	6 (37.50%)
No, *n* (%)	8 (50.00%)
Unsure, *n* (%)	2 (12.50%)
Completed Supplementary Online Training Video Capsules	
Yes, *n* (%)	3 (18.75%)
No, *n* (%)	10 (62.50%)
Unsure, *n* (%)	1 (6.25%)
No response, *n* (%)	2 (12.50%)
Accessed *Super Splendide*’s Technical Support	
Yes, *n* (%)	4 (25.00%)
No, *n* (%)	8 (50.00%)
Unsure, *n* (%)	2 (12.50%)
No response, *n* (%)	2 (12.50%)
Number of VR sessions facilitated per month, *Mean* (*SD*)	1.50 (1.21)
VR session length in minutes, *Mean* (*SD*)	17.69 (14.30)
Hours spent facilitating VR over the past 6 months, *Mean* (*SD*)	5.69 (7.75)
Number of residents using VR per session, *Mean* (*SD*)	2.69 (1.70)

All training sessions were provided by *Super Splendide* via its VR training curriculum. VR = virtual reality.

### 3.2 Quantitative findings

This section presents descriptive statistical findings from the VR-use questionnaire, grouped together by DTPB domains.

#### 3.2.1 Attitudes (general attitudes, perceived usefulness, perceived ease of use, compatibility)

**Figure 1:**
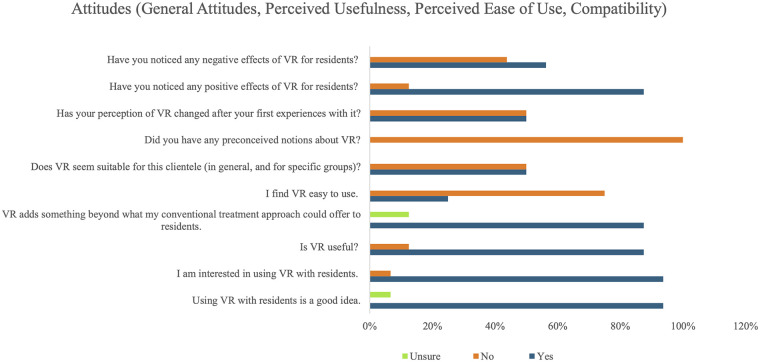
LTC staff's responses (*n* = 16) to the modified VR-Use questionnaire with statements targeting attitudes (overall, perceived usefulness, perceived ease of use, and compatibility). “Residents” refers to older adult residents of Long-Term Care (LTC) facilities; VR, Virtual Reality.

Participants had generally positive attitudes about using immersive VR, with 93.8% (*n* = 15) endorsing it as a good idea to use with LTC residents, 87.5% (*n* = 14) indicating that they found it useful, and 87.5% (*n* = 14) reporting that VR added something to their conventional treatment approach. However, only 25.0% (*n* = 4) found VR easy to use, with 50.0% (*n* = 8) of participants indicating that they found it suitable for LTC clientele. Similarly, while 87.5% (*n* = 14) of LTC staff reported noticing positive effects of VR for residents, another 56.3% (*n* = 9) indicated that they had also observed negative effects when using VR with residents. Further information on attitudes towards VR use can be found in Figure 1.

#### 3.2.2 Social norms (peer influence, client influence, superior influence)

**Figure 2:**
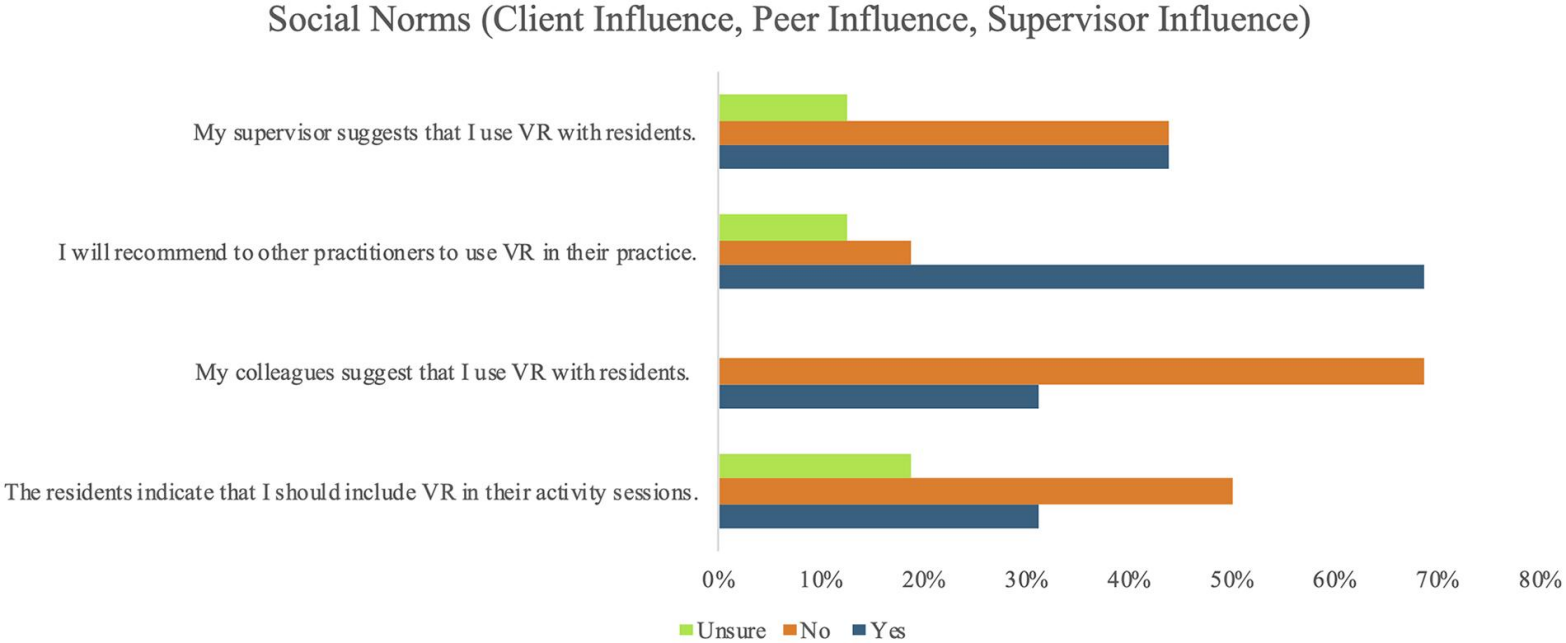
LTC staff's responses (*n* = 16) to the VR-Use questionnaire with statements targeting social norms (client influence, peer influence, and supervisor influence). “Residents” refers to older adult residents of Long-Term Care (LTC) facilities; VR, Virtual Reality.

Participants reported mixed findings with respect to social norms for VR use [i.e., key factors shaping LTC staff behavior based on what they believe peers, supervisors, and residents approve of (66)]. They indicated receiving limited influence from residents (31.3%; *n* = 5) and peers (31.3%; *n* = 5) to use VR in their practice. A slightly larger proportion of participants reported being influenced to use VR by their supervisors (43.8%; *n* = 7). In contrast, 68.8% (*n* = 11) indicated that they would recommend VR use to others in their practice. Further information on the social norms' domain can be found in Figure 2.

#### 3.2.3 Perceived behavioural control (self-efficacy, behavioural intention)

**Figure 3:**
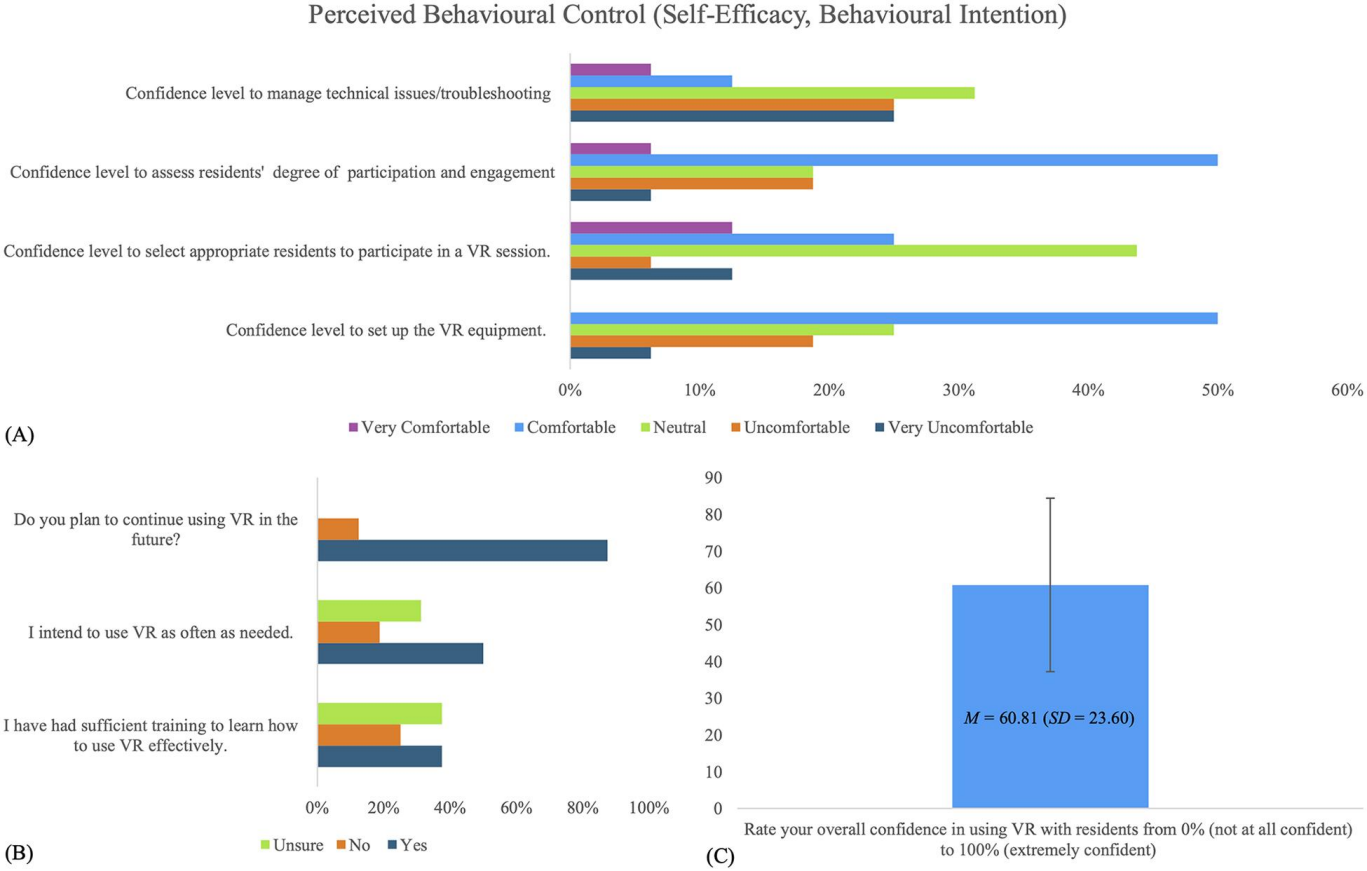
LTC staff's responses (*n* = 16) to the modified VR-Use questionnaire with statements targeting perceived behavioural control (self-efficacy, and behavioural intention). **(A)** Confidence level with VR; **(B)** intention to use VR; **(C)** overall confidence with VR use. “Residents” refers to older adult residents of Long-Term Care (LTC) facilities; VR, Virtual Reality.

Regarding perceived behavioural control in VR use, overall participants reported feeling moderately comfortable using VR with residents (*M* = 60.81, *SD* = 23.6) when asked to rate their confidence on a 0–100 scale (0 = lowest confidence). However, their comfort level varied depending on the specific task. LTC staff felt relatively comfortable assessing residents' participation and engagement (56.3%, *n* = 9) and setting up the VR equipment (50.0%, *n* = 8). In contrast, they expressed lower confidence in selecting appropriate residents for VR activities, with 37.5% (*n* = 6) of participants reporting that they were comfortable doing this. Similarly, only 18.8% (*n* = 3) of participants reported confidence with respect to troubleshooting technical issues. In line with these findings, 37.5% (*n* = 6) of participants indicated that they have received enough training to effectively use VR. Despite lower reported levels of confidence in their training and practice, 87.5% (*n* = 14) of participants expressed that they intended to continue using VR in their future work. Further details on perceived behavioural control can be found in Figure 3.

#### 3.2.4 Facilitating conditions and barriers

Overall, participants seemed to highlight barriers as being more prevalent than facilitators. The most highly endorsed facilitator to VR use in LTC was having adequate space (93.8%, *n* = 15). Additionally, some participants indicated that residents who had already been offered VR sessions appeared to be motivated to continue using the technology (56.3%; *n* = 9). Half of participants (*n* = 8) indicated feeling adequately supported by management to use VR in their work. However, the remaining 50.0% reported feeling either not well-supported (31.3%; *n* = 5), or unsure of their support from management (18.8%; *n* = 3).

**Figure 4:**
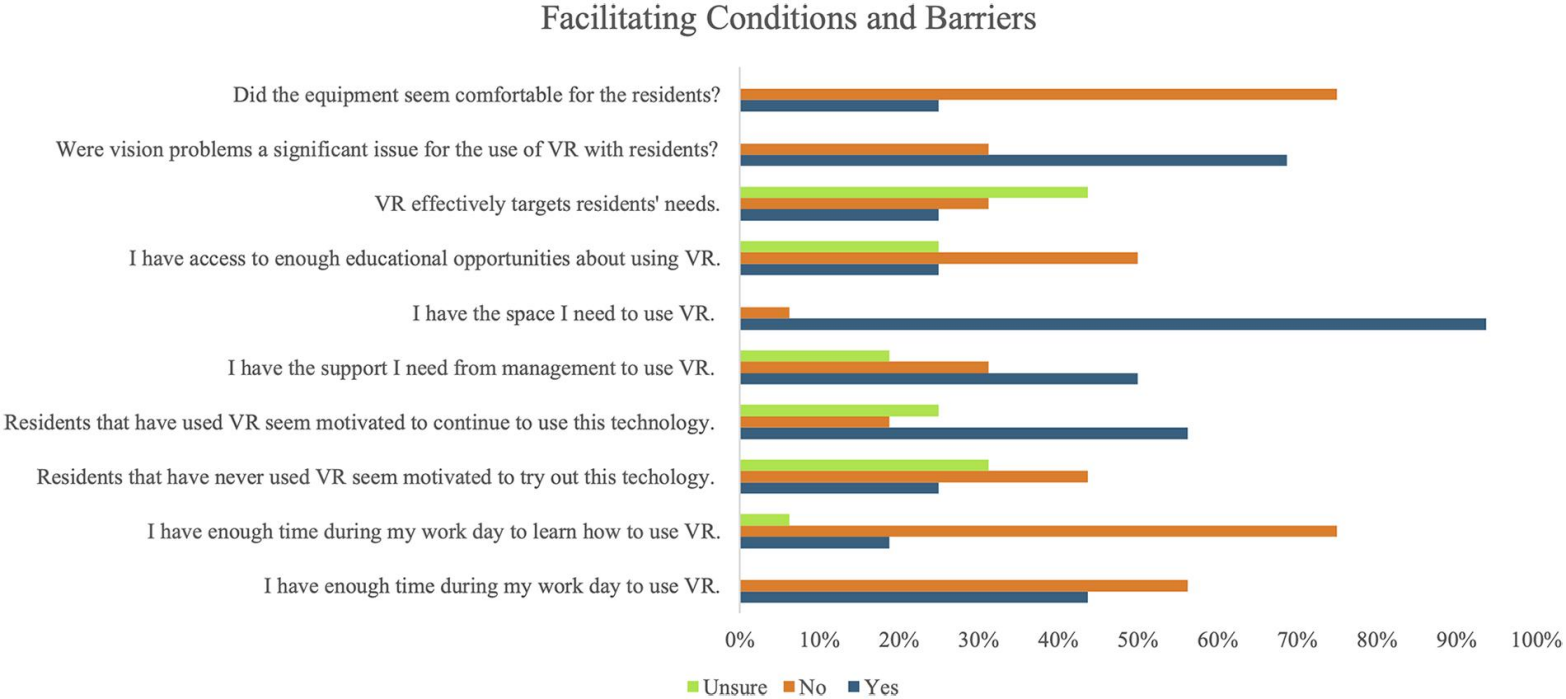
LTC staff's responses (*n* = 16) to the modified VR-Use questionnaire with statements targeting facilitating conditions (barriers and facilitators). “Residents” refers to older adult residents of Long-Term Care (LTC) facilities; VR, Virtual Reality.

Additional barriers of note included lack of time to both use VR during the workday (56.3%; *n* = 9) and learn *how* to use it (75.0%; *n* = 12), residents' discomfort with equipment (75.0%; *n* = 12) and vision issues (68.8%; *n* = 11) during VR use, and lack of clarity with regards to whether VR effectively targets the needs of residents (43.8%; *n* = 7 reported being unsure, 31.3%; *n* = 5 reported it was ineffective). Additional information on barriers and facilitators can be found in Figure 4.

#### 3.2.5 Perspectives on VR training received

In general, LTC staff found the VR training they received from *Super Splendide* helpful. Participants on average reported that the training quality was high when asked to rate it on a scale from 0 to 100 (*M* = 77.50; *SD* = 16.82). However, they also noted that the training had room for improvement with 50.0% (*n* = 8) of participants noting that it could be strengthened, when asked. The discrepancies in positive attitudes towards training and requested improvements in the education they received are explored further in the qualitative findings.

### 3.3 Qualitative findings

A total of 414 meaning units were drawn from participants' verbatim responses and were organized into 97 deductive and inductive code groupings, with their respective subcodes. These codes were then synthesized into nine distinct subcategories, which in turn were consolidated into four higher-order categories, informing the development of the final themes. Overall, coding decisions between coders were highly consistent: only 38 coding decisions were modified, 10 of which were resolved by a third party. The following four themes were generated regarding staff's perspectives on VR use in LTC:

1. When barriers outweigh promise: Challenges undermining the sustainable integration of VR in LTC.
2. Personalizing the VR experience: Embracing an individualized perspective to enhance care provision.
3. Optimizing the experiential environment and organizational structures: Building the conditions for sustainable VR use with residents in LTC.
4. Ambivalence in practice: Staff weigh promise and pragmatics when adopting VR in care facilities.

Findings are presented by theme, organized into subcategories, and include definitions, pertinent participant quotations, and linkages to relevant DTPB constructs. See Table 3 for a summary of qualitative themes, categories, sub-categories, and representative quotes.

**Table 3:** Qualitative themes, categories, subcategories, and representative participant quotes.

Themes	Categories	Subcategory	Pertinent DTPB Constructs	Representative Quotes
When barriers outweigh promise: Challenges undermining the sustainable integration of VR in LTC	Challenges and obstacles related to the use of VR in LTC	Resident-related challenges and obstacles to the use of VR in LTC	Attitudes—Compatibility; Social Norms—Client Influence; Perceived Behavioural Control—Self-Efficacy; Facilitating Conditions—Barriers	*“Wearing glasses sometimes seems to be a problem. It is difficult to know if the person can see properly, as the quality of the image can be affected by how the headset is worn.” (PID-08)*
		Challenges and obstacles related to technology and the usability of VR in LTC	Attitude—Perceived Ease of Use; Facilitating Conditions—Barriers	*“Residents do not express willingness to participate in virtual reality activities.” (PID-19)*
		Challenges and obstacles related to staff knowledge of VR in LTC	Perceived Behavioural Control—Self-Efficacy; Facilitating Conditions—Barriers	*“The headsets we have at our LTC facility aren*’*t the latest version and have velcro head straps. They pull on residents*’ *hair, and the headsets are very heavy.” (PID-10)*
				*“Difficult to connect to the Wi-Fi, which affects the quality of certain videos” (PID-16)*
				*“I don*’*t have enough time to properly understand how VR works to be able to use it well with residents” (PID-17)*
				*“Training was a long time ago. If the system isn*’*t used every week, you forget it.” (PID-18)*
Personalizing the VR experience: Embracing an individualized perspective to enhance care provision	Individualized needs and benefits of using VR in LTC	Resident selection and personalization of the VR experience based on individualized needs in LTC	Attitude—Compatibility, Perceived Usefulness, Perceived Ease of Use	*“Older adults with loss of autonomy and cognitive decline will need close supervision, go slowly” (PID-10)*
		Personalized benefits of using VR for residents in LTC	Attitude—Perceived Usefulness; Perceived Behavioural Control—Behavioural Intention	*“Try to connect with their interests in order to motivate them to perform physical activities (climbing stairs, playing golf, hunting, walking, etc.), get involved in an activity (household chores), and create moments of calm (exploring their hometown or village, traveling, etc.). Build trusting relationships by experiencing successes. Respond to their unmet emotional/sexual needs.” (PID-13)*
				*“Some people who are usually unresponsive have shown positive reactions. It sparks discussion and curiosity” (PID-08)*
				*“Relaxation, decreased BPSD, enhanced self-esteem, less boredom, and fewer daytime naps” (PID-17)*
Optimizing the experiential environment and organizational structures: Building the conditions for sustainable VR use with residents in LTC	The experiential environment and its associated needs and challenges	Staff needs related to the implementation and use of VR in real-world settings	Facilitating Conditions—Facilitators; Social Norms—Peer Influence; Supervisory Influence	*“How to prepare before a VR session/have the necessary recreation staff available?” (PID-14)*
		Organizational challenges related to the implementation and use of VR in real-world settings	Attitude—Compatibility, Perceive Ease of Use; Facilitating Conditions—Barriers	* “To be able to get additional support directly in our LTC centres” (PID-07)*
				*“The more we put it into practice, the more comfortable we*’*ll be and the more interested the residents will become.” (PID-14)*
				*“takes too much time for the [number of] people reached by the activity” (PID-01)*
				*“Lack of time, I have to take it home on my personal time to practice with it.” (PID-18)*
Ambivalence in practice: Staff weigh promise and pragmatics when adopting VR in care facilities	Attitudes toward technology and training	Positive attitudes toward technology and training	Attitude –Perceive Ease of Use, Compatibility; Perceived Behavioural Control—Behavioural Intention, Self-Efficacy	*“The cognitive, sensory, and emotional aspects: I consider it to be truly* ‘*a journey*’*” (PID-10)*
		Negative and/or ambivalent attitudes toward technology and training	Attitude—Perceive Ease of Use, Compatibility; Perceived Behavioural Control –Self-Efficacy; Facilitating Conditions—Barriers	*“Often prisoners of their own bodies, so this lets them escape for a moment.” (PID-02)*
				*“Provides us with another tool in our toolbox as practitioners, enabling us to help as many residents as possible” (PID-17)*
				*“In my opinion, it doesn*’*t reach a large enough number of our clientele” (PID-06)*
				*“Feeling alone in the world. No one else is trained.” (PID-18)*

DTPB, Decomposed Theory of Planned Behaviour; VR, Virtual Reality; LTC, Long-Term Care.

#### 3.3.1 Theme 1—when barriers outweigh promise: challenges undermining the sustainable integration of VR in LTC

This theme captured the technological, resident-related, and staff-specific obstacles that overshadowed the perceived benefits of VR, potentially limiting its sustainable integration and use in LTC. Participants described practical constraints such as lacking the time necessary to learn how to use VR, technical difficulties, and compatibility issues with residents' functional realities. Three subcategories were identified in relation to barriers in VR use in LTC: (1) Resident-related challenges and obstacles to the use of VR in LTC, (2) Challenges and obstacles related to technology and usability of VR in LTC, and (3) Challenges and obstacles related to staff knowledge of VR in LTC.

##### 3.3.1.1 Theme 1.1—resident-related challenges and obstacles to the use of VR in LTC

Staff described resident profiles as decisive for VR feasibility, noting that advanced neurocognitive impairment, visual limitations, and discomfort with headsets could curtail engagement even when interest was present. Staff frequently highlighted cognitive impairment as a barrier to resident engagement with VR programming [Attitudes—Compatibility], voicing that “*Most residents have neurocognitive disorders, so they don’t understand. They often refuse to even put on the headset”* (PID-01) and noting that this made it difficult for them to know who to recruit when organizing VR activities [Social Norms—Client Influence]: “*Choosing our candidates. It's not easy to find participants for whom we can feel a positive effect”* (PID-06). Furthermore, difficulties with headset discomfort [Facilitating Conditions—Barriers] were highlighted: “*The headset is heavy on the head; some don’t want to keep it on”* (PID-04). In addition, challenges related to residents’ vision and communication regarding the quality of the VR image [Perceived Behavioural Control—Self-Efficacy] were identified: “*Not knowing their visual capacity, pain, dryness, blurred vision, glaucoma, macular degeneration, or other issues: we can assume they see well, but we have no certainty”* (PID-10).

##### 3.3.1.2 Theme 1.2—challenges and obstacles related to technology and usability of VR in LTC

Hardware fit and comfort, connectivity stability, and interface complexity were recurrent friction points that diminished ease of use and staff confidence during sessions. Staff frequently mentioned challenges with internet connectivity and Wi-Fi as barriers to VR usability in LTC and to the image quality [Attitude—Perceived Ease of Use]: “*Difficult to connect to the Wi-Fi, which affects the quality of certain videos*” (PID-16)*.* Additionally, elements of the headset hardware and related unwieldiness were highlighted as decreasing the quality of the VR experience, such as the velcro headset strap [Facilitating Conditions—Barriers]: “*Better [headset] strap to make it more comfortable”* (PID-18). Furthermore, staff reported that the complexity of the VR software and its interface could also impede its usability [Attitude—Perceived Ease of Use]: “*The interface is difficult to set-up”* (PID-07), and noted learning curves to accessibility “*Need to know how to use technology skillfully”* (PID-014).

##### 3.3.1.3 Theme 1.3—challenges and obstacles related to staff knowledge of VR in LTC

Staff confidence was constrained by uneven or insufficient training, lack of familiarity with the hardware/software and its related protocols, uncertainty about safety steps, and over-reliance on “super-users”. Elements such as training deficits [Facilitating Conditions-Barriers] were emphasized “*I lack basic training on how to use the equipment*” (PID-18), as well as limited time during the workday to practice and consolidate what was learned during training sessions [Perceived Behavioural Control—Self-Efficacy] “*I don’t have enough time to properly understand how VR works to be able to use it well with residents”* (PID-17). Participants also voiced insecurity with running the VR systems on their own [Perceived Behavioural Control—Self-Efficacy] “*I don’t know how to use all the controls”* (PID-14) and feeling burdened to help other staff members who had not been trained “*[their colleague] didn’t receive the same training… has to wait for other colleague who has been trained”* (PID-13). Despite having completed initial training sessions, participants' feedback suggests that one-off training workshops were not sufficient to maintain confidence with VR over time, particularly without opportunities for continued practice or access to real-time support.

#### 3.3.2 Theme 2—personalizing the VR experience: embracing an individualized perspective to enhance care provision

This theme reflected participants' emphasis on tailoring VR interventions to LTC residents' personal histories, preferences, and needs. Individualization was viewed as key to fostering engagement, emotional resonance, and therapeutic value. Staff perspectives connected to this theme highlighted how VR could move beyond a one-size-fits-all activity to become a more meaningful part of person-centred care. Two subcategories were captured in connection to this theme: (1) Resident selection and personalization of the VR experience based on individualized needs in LTC, and (2) Personalized benefits of using VR for residents in LTC.

##### 3.3.2.1 Theme 2.1—resident selection and personalization of the VR experience based on individualized needs in LTC

This subcategory emphasized targeted selection and tailored content as prerequisites for suitability of VR activities. Pacing and close support were also emphasized for residents with cognitive and sensory limitations. Staff highlighted the value of developing a personal understanding of residents, which could then be applied to their selection of VR content [Attitude—Compatibility]: “*Know the person's tastes and interests, and then find videos that may interest them”* (PID-10), with many mentioning the utility of personalizing VR videos to residents' places of birth and developing activities connected to these settings [Attitude—Perceived Usefulness] “*Have our residents travel to their own neighborhoods. Get them to exercise through tasks they know… milking a cow, chopping wood”* (PID-16). Additionally, staff expressed the necessity of tools that mirror the headset view to help them better guide the residents' activities [Attitude—Perceived Ease of Use] “*To be able to have access to what the resident is seeing at the same time as them…* via *the tablet, without having to remove the headset from the resident”* (PID-13), and highlighted the importance of remembering the specific needs of the clientele that they work with when selecting residents for VR activities [Attitude—Compatibility] “*Older adults with loss of autonomy and cognitive decline will need close supervision, go slowly”* (PID-10).

##### 3.3.2.2 Theme 2.2—personalized benefits of using VR for residents in LTC

When well-matched to residents, staff described VR as eliciting moments of awe, reminiscence, and calm. Some participants also described observing reductions in behavioural and psychological symptoms of dementia (BPSD). Many staff highlighted VR's potential to create feelings such as awe and happiness through its immersive qualities [Attitude—Perceived Usefulness]: “*Most smile and exclaim that it's incredible”* (PID-10). Furthermore, participants highlighted VR's ability to elicit curiosity and engagement in typically non-responsive residents [Attitude—Behavioural Intention]: *“Some people who are usually unresponsive have shown positive reactions. It sparks discussion and curiosity”* (PID-08), as well as its ability to increase residents' healthy functioning [Attitude—Perceived Usefulness]: “*Relaxation, decreased BPSD, enhanced self-esteem, less boredom, and fewer daytime naps”* (PID-17).

#### 3.3.3 Theme 3—optimising the experiential environment and organizational structures: building the conditions for sustainable VR use with residents in LTC

This theme explores how the physical/sensory milieu (e.g., space, lighting noise, privacy, mobility supports) and the organizational architecture (e.g., workflows, staffing, equipment access, training, protocols, leadership buy-in) interact to either enable or obstruct VR delivery in LTC. Participants emphasized the necessity of deliberate planning to optimize VR activities in order to normalize it as part of routine, person-centred care rather than it being viewed an interesting but unessential add-on. Two subcategories were generated in connection to this theme: (1) Staff needs related to the implementation and use of VR in real-world settings, and (2) Organizational challenges related to the implementation and use of VR in real-world settings.

##### 3.3.3.1 Theme 3.1—staff needs related to the implementation and use of VR in real-world settings

This subcategory referred to the concrete resources, competencies, and professional supports required to integrate VR into routine practice. Staff emphasized two key enabling factors. First, they called for mandatory, standardized training [Social Norms—Peer/Supervisor Influence]: “*Training should be mandatory for staff… to ensure optimal use*” (PID-13). Second, they asked for clearly identifiable supports and on-site coaching to help with troubleshooting and better matching of content to residents [Social Norms—Peer/Supervisor Influence]: “*Make access available to a point person to guide us based on the resident's individualized needs*” (PID-13). Additionally, the need for access to settings conducive to VR sessions was highlighted [Facilitating Conditions—Facilitators]: “*[Need a] room that's less noisy”* (PID-16), “*It's very important to turn off the lights and close the curtains to prepare [their] vision*” (PID-10).

##### 3.3.3.2 Theme 3.2—organizational challenges related to the implementation and use of VR in real-world settings

Staff reports indicated that it was difficult for sites to reconcile one-on-one VR sessions with group programming and limited staff time, pointing to a pattern where VR delivery was constrained due to organizational structures and workflows. Factors such as set-up and scheduling were frequently cited barriers to VR usage [Facilitating Conditions—Barriers] “*The time it takes to prepare and set up is too long”* (PID-06), with some staff expressing that the benefits of VR-based activities were not worth the amount of effort they took to set-up [Attitude—Perceived Usefulness]: “*takes too much time for the [number of] people reached by the activity”* (PID-01), and noting that they found it difficult to run group-based VR activities [Attitude—Compatibility]: “*I can’t include it in my group activities”* (PID-18). Furthermore, some mentioned the added difficulty of bureaucratic documentation taking away from their time directly engaging in activities with residents [Facilitating Conditions—Barriers]: “*Fewer ministerial constraints, less paperwork, and more time on the floor”* (PID-09).

#### 3.3.4 Theme 4—ambivalence in practice: staff weigh promise and pragmatics when adopting VR in care facilities

This theme expands upon LTC staff attitudes to VR use, highlighting a pattern of ambivalence. Despite excitement about VR's potential to engage residents and enrich care, there is skepticism rooted in workload pressures, technological reliability, resident suitability, and the perceived “value-add” of VR. Responses ranged from strong enthusiasm and cautious curiosity to measured resistance, shifting with the availability of support and training, and with how visible resident benefits were. Two subcategories were identified in relation to this theme: (1) Positive attitudes toward technology and training; (2) Negative and/or ambivalent attitudes toward technology and training.

##### 3.3.4.1 Theme 4.1—positive attitudes toward technology and training

Participants described how positive prior experiences, confidence with technology, and alignment with person-centred care positively shaped their stance regarding VR use. Staff voiced how hands-on successes and visible resident benefits nurtured positive appraisals [Attitude—Perceived Usefulness]: “*I've had wonderful experiences with people I never would have expected”* (PID-08). Participants also mentioned how their positive experiences were linked to openness to continued use of VR in LTC [Perceived Behavioural Control—Behavioural Intention]: “*[I will] include it in my schedule every month!! My goal is to do at least one [session] every week = four per month”* (PID-10). In line with these findings, confidence using VR was also emphasized as a factor contributing to continued use [Perceived Behavioural Control—Self Efficacy]: “*I am confident that I have the skills and knowledge to use virtual reality”* (PID-06), in addition to offering high-quality person-centred care [Attitude-Compatibility] “*Provides us with another tool in our toolbox as practitioners, enabling us to help as many residents as possible”* (PID-17).

##### 3.3.4.2 Theme 4.2—negative and/or ambivalent attitudes toward technology and training

Staff voiced skepticism tied to set-up complexity, time pressure, and training gaps (e.g., coverage, training recency, limited troubleshooting practice). For instance, participants mentioned the lack of fit of the apps available on the Meta VR headsets with their clientele [Attitude—Compatibility]: “*the games and applications available on the Meta Quest store are not well adapted to our residents’ needs”* (PID-17). They also noted continued challenges with VR's usability [Attitude—Perceived Ease of Use]: “*Difficult to use with people who have major neurocognitive disorders”* (PID-01). Further contributing to staff's reported negative or ambivalent feelings to VR use were impressions that they either lacked training or time to practice the skills that they had learned were necessary to facilitate VR sessions [Facilitating Conditions—Barriers]: “*Often hard to put into practice… lack of time, no training”* (PID-13), and noted deterioration of competence [Perceived Behavioural Control—Self-Efficacy] “*however, due to lack of use, I’ve forgotten several concepts”* (PID-13).

### 3.4 Integration of quantitative and qualitative findings (mixed-methods analysis)

Across qualitative themes and quantitative results, LTC staff appeared to judge immersive VR as valuable in principle, but routine use seems to be conditional on resident-technology fit and ease-of-use at the point of care. Favorable attitudes coexisted with only moderate confidence, especially with respect to quantitative results on resident selection and troubleshooting. Qualitative accounts tied these weak points to time and workflow pressures, connectivity/interface issues, headset comfort, and a narrow content library. Conversely, individualized sessions delivered in calm spaces often yielded perceived resident engagement, reminiscence, calm, and occasionally reduced BPSD, pointing to potential of VR use in LTC settings, especially when conditions and delivery are optimized. Adoption strengthened where facilitating conditions (e.g., protected time, headset mirroring for real-time guidance, on-unit support, broader culturally relevant content, hardware upgrades) and social infrastructure (e.g., visible leadership backing, peer modeling, multiple trained staff per unit) were in place. These facilitators in turn appear to bolster self-efficacy, potentially helping to normalize VR as “real care”.

The integration meeting also clarified several measurement nuances. First, “adequate space” for VR sessions (endorsed by 93.75%) was interpreted qualitatively as environment quality (quiet, dimmable, private), suggesting that an item assessing space needs with only limited response options may overstate feasibility, unless sensory conditions are specified. Second, several “don’t know” responses (e.g., supervisor support) were interpreted with respect to the LTC context. For example, top-down rollouts of VR technology by regional managers sometimes left ambiguity during unit-level implementation, helping to explain mixed social-influence signals, despite generally positive attitudes reported by participants. Finally, wording such as “as often as necessary”, and limited prior exposure to VR, likely contributed to ambivalence on some behavioural intention items on the questionnaire. The value of tailoring some items by experience level and clarifying definitions (e.g., “session”) was highlighted for future studies by team members.

In summary, staff uptake appeared to rise when compatibility, ease-of-use, enabling conditions, and social support align. Findings also pointed to the potential for ambivalence to resurface and VR use to taper off when any of the aforementioned facilitators were absent. See Table 4 for further details on integration of quantitative and qualitative findings.

**Table 4:** Joint display of integrated qualitative and quantitative findings on VR adoption in long-term care.

DTPB construct	Key quantitative findings (*n* = 16)	Qualitative pattern	Integrated meta-inference	Practical implication/action	Relation between strands (Confirmation/Expansion/Discordance)
Attitude (Perceived Usefulness & Compatibility)	VR is a good idea: 93.75%; VR is useful: 87.50%; VR adds to usual approach: 87.50%; VR suitable for LTC clientele: 50.00%.	Value strongest when content is personalized and calming; benefits include awe, reminiscence, reduced BPSD. e.g., “*Most smile and exclaim that it*’*s incredible”* (PID-10); “*Relaxation, decreased BPSD…”* (PID-17)	Positive appraisals are conditional on resident–technology fit; when matched to history/preferences, usefulness is salient.	Curate resident-matched content; brief pre-session preference check; maintain a longer, culturally relevant content library.	Expansion
Attitude (Perceived Ease of Use)	VR easy to use: 25.00%; Neutral to low comfort with troubleshooting: 81.25%	Usability barriers: Wi-Fi instability, blocked apps, interface complexity, headset comfort. e.g., “*Difficult to connect to the Wi-Fi”* (PID-16); “*The headset is heavy on the head”* (PID-04)	Constraints to ease-of-use helped explain why adoption remained modest despite positive attitudes	Enable reliable screen-mirroring; quick-reference troubleshooting guides; pre- downloaded content; upgrade headset straps; test Wi-Fi on unit.	Confirmation
Perceived Behavioural Control (Self-Efficacy & Behavioural Intention)	Overall confidence level for VR use, from 0 to 100: *M* = 60.81 (*SD* = 23.6); Comfortable assessing resident engagement: 56.25%; Confident in VR set-up: 50.00%; Neutral/ low comfort selecting residents: 62.50%; Feel sufficiently trained: 37.50%; Intend continued VR use: 87.50%	Confidence declines without practice; limited time to learn; feeling “alone” as the only trained user. e.g., “*Training was a long time ago… you forget it.”* (PID-18); “*Lack of time”* (PID-12)	Confidence is brittle at selection and troubleshooting; targeted refreshers and shared expertise are needed for routine use.	Regular, short, hands-on refreshers; scenario-based practice; designate a unit “VR champion”.	Confirmation
Social Norms (Peer & Supervisor Influence)	VR use suggested by peers: 31.25%; VR use suggested by residents: 31.25%; VR use suggested by supervisor: 43.75%; Would recommend VR: 68.75%	Optimism for VR’s potential, but day-to-day use depends on local support; limited peer modelling at sites where only one person is trained. e.g., « “*Training should be mandatory…”* (PID-13); “*Step-by-step guides”* (PID-17)	Visible leadership endorsement and peer modelling are essential to normalize VR as “real care”.	Communicate clear leadership support during training; organize brief peer-led demos; expand training to multiple staff per unit.	Confirmation
Facilitating Conditions (Barriers & Facilitators)	Facilitators: Adequate space: 93.75%; Resident motivation: 56.25%. Barriers: Lack of time to use: 56.25%; Lack of time to learn 75.00%; Resident discomfort: 75.00%; Vision issues: 68.75%); Supported by management: 50.00%	Needs: quiet/dedicated spaces; better straps; reliable screen mirroring; broader content diversity; on-site support. e.g., “*room that*’*s less noisy”* (PID-16); “*Have more videos of places to visit; be able to easily share the image on the TV”* (PID-08)	Implementation is constrained more by workflow, time, and infrastructure than by perceived value of VR itself.	Protected time blocks for VR; create set-up checklists; pre-test room/equipment; provide on-unit real-time support; expand content length and diversity.	Expansion/Discordance

DTPB, Decomposed Theory of Planned Behaviour; VR, Virtual Reality; LTC, Long-Term Care; BPSD/SCPD, Behavioural and Psychological Symptoms of Dementia. “Confirmation” refers to both types of data (i.e., quantitative, qualitative) confirming the results of the other; “Expansion” refers to findings from two types of data diverging but covering complementary facets of the same phenomenon, broadening understanding; “Discordance” refers to inconsistencies, contradictions, or disagreements between qualitative and quantitative findings.

## 4 Discussion

This convergent, cross-sectional mixed-methods study examined LTC staff's adoption of immersive VR, following the DTPB framework to focus on how attitudes, social norms, perceived behavioural control, and facilitating conditions shape day-to-day use. Our results identified where adoption succeeds or stalls (e.g., selection and troubleshooting), when benefits are most salient (e.g., personalized content in a calming experiential environment), and which changes are most feasible and needed (e.g., time blocks, support, training, content, ergonomics). Together, these insights define a practical implementation roadmap for VR in LTC. When delivered under the identified conditions, VR may serve as a low-burden, person-centred activity that promotes relaxation, positive mood, reminiscence, and engagement. These factors were identified by staff as relevant for residents' well-being and quality-of-life. Findings from this research are thus intended to inform the design and implementation of future VR-based interventions that are feasible, person-centred, and sustainable in real-world LTC. Below, we discuss barriers, facilitators, potential strategies, and current developments for VR adoption in the LTC context, organized by the themes generated during data analysis.

### 4.1 When barriers outweigh promise

Staff endorsed VR's value “in principle’, but routine use appeared to falter at the points of resident selection and technical troubleshooting. These difficulties, specifically selecting appropriate residents for VR activities and troubleshooting technical issues, were evident in the quantitative data, where only 37.5% of participants reported confidence in resident selection and 18.8% in troubleshooting. Qualitative findings further explained these challenges by highlighting time and workflow pressures, connectivity constraints, interface complexity, and headset comfort issues. Our results align with Wong and colleagues (54), who identified conditions under which staff were more likely to integrate VR into routine care. In line with the DTPB, our findings extend this work, and indicate that long-term integration of new technologies in LTC is unlikely when staff do not feel adequately resourced to deliver VR and perceive limited acceptability among residents. Given the potential benefits that VR can offer, our study highlights the need to explore solutions that improve user-friendliness for LTC staff and enhance the experience for residents, based on staff perceptions of what residents found acceptable and engaging. Suggested areas to enhance usability and usefulness in the literature include codeveloping VR implementation toolkits with LTC staff to facilitate troubleshooting, workflows, content selection and headset hygiene (54, 56, 81), mitigating headset discomfort for residents by selecting lighter devices with clear visuals and careful motion design (28, 54, 56, 82), and simplifying session involvement for residents through shorter sessions, seated use, simple headset controls, and staff facilitation (28, 54–56, 82).

Accordingly, our research team has been incorporating these suggestions into practice with the care teams at the CISSS-CA. As part of this process, data from our study highlighted a major issue related to comfort and usability of immersive VR headsets for older adults in the CISSS-CA context. VR participants frequently reported discomfort due to poorly adapted straps and the considerable weight of the devices. For example, the Meta Quest 2 with the Elite Strap can weigh as much as 550 g, proving burdensome for residents with limited cervical strength and endurance, as observations indicated that some residents could not maintain their head upright under this weight load. By contrast, recent devices demonstrate significant progress in miniaturization and weight reduction. The HTC Vive Flow (83) weighs 189 g, and the Bigscreen Beyond 2 (84) currently stands as the lightest immersive VR headset on the market at only 107 g, illustrating the potential for major improvements in ergonomics. As the Quest 2 headsets were already implemented in the CISSS-CA's care environments, our team developed an articulated arm system to mitigate discomfort associated with the weight of the headset.

### 4.2 Personalizing the VR experience

Integrated findings from this study suggest that VR usefulness is likely conditional upon compatibility, which may be enhanced through individualized content (i.e., aligned with residents' histories, preferences, and sensory needs) delivered in calm spaces. LTC staff perceived these conditions as being linked to resident engagement, reminiscence, relaxation, and occasional reductions in BPSD. Consistent with recommendations by Chaze et al. (56) and Orr et al. (28), to prioritize calming and familiar VR content, such as 360-degree nature, relaxing audio scripts, or travel experiences, our findings indicate that LTC staff observed relaxation and reminiscence benefits for residents during VR activities with nature videos.

Furthermore, our results support that tailoring VR content to residents’ preferences and needs may improve engagement and outcomes. Staff highlighted the importance of fitting technology to residents' current level of functioning and matching content to their past experiences. This finding parallels research emphasizing the important of co-designing VR programs to reflect older adults' preferences and identities, and personalizing scenarios through the use of elements that enhance user immersion and appreciation, such as local environments, culturally-meaningful audio, and family photos (53, 56, 82). This study adds an implementation-focused perspective to the existing literature by showing, in a real-world LTC context, how personalization aligns with DTPB constructs that shape staff uptake (i.e., compatibility, perceived usefulness/ease of use, and self-efficacy) and offering practical targets such as content match, session pacing, and delivery context, to promote sustainable VR use in routine LTC care.

### 4.3 Enabling conditions: environment and organization

Our findings converged on the perceived necessity of environmental and organizational factors to enable VR to become a routine part of person-centred care. Facilitating conditions such as dependable access to online content, on-site technological help, standardized training with more than one staff member trained per site, adequate support from management, and sufficient time to learn how to use VR were frequently mentioned factors to promote better integration into LTC settings. As per the DTPB, these conditions align with staff's perceived ease of use, self-efficacy, and social norms via supervisor and peer influence, ultimately promoting the conversion of favourable attitudes into intention and use. They also align with implementation guidance from the scientific literature citing the benefits of appointing on-site “champions’, providing ongoing training, planning for Wi-Fi instability, and securing leadership buy-in for protected time and equipment (53, 54, 57, 81).

At the organizational level, staff described structural barriers typical to LTC, such as staffing pressures and lack of dedicated time. This finding reinforces the importance of management support, availability of ongoing training refreshers, and shared VR skill coverage between multiple professionals to prevent the reliance on a single “VR person” and the feelings of isolation that this staff member might feel. Our results are consistent with review articles calling for theory-informed implementation that moves beyond the surface identification of barriers by suggesting explicit strategies to improve VR adoption, such as training modifications, on-site champions, workflow integrations, and technical support (53, 57). Taken together, our findings may help to operationalize VR as a low-burden intervention for pain-relevant care, by making it more feasible for staff to deliver calming, individualized sessions that support relaxation and safe engagement in routine practice.

### 4.4 Ambivalence toward VR among staff

Ambivalence towards VR use in LTC appeared to be relatively common for staff when ease-of-use and facilitating conditions lagged, even with the presence of initially positive attitudes. Participants noted enthusiasm about VR's potential to engage residents, while concurrently voicing skepticism connected to workload pressures, technological reliability, resident suitability, and uncertainty about VR's contribution beyond usual care. Quantitatively, favourable attitudes were paired with only moderate perceived behavioural control, especially for resident selection and troubleshooting, as previously mentioned. These findings have also been raised in studies focused on VR use for post-stroke recovery (85). This overlap suggests that, although context-specific factors drive implementation, cross-cutting psychological factors (e.g., perceived usefulness of technology) also matter, but may not be enough to sustain VR adoption across diverse care settings. Qualitatively, difficulties were attributed to factors such as time constraints, internet connectivity issues, and technology complexity, leading to increases in perceived barriers and decreases in ease-of-use from staff's point-of-view. These perceived challenges are connected to theoretical and empirical evidence predicting reduced intention to use VR despite otherwise positive attitudes (53, 63, 66). In the broader LTC context, such ambivalence appears to be common due to the prevalence of constraints limiting consistent access to enabling conditions (53, 81).

Staff ambivalence was mitigated when support and visibility were strong. For example, residences with more than one facilitator trained to use VR, improvements to standard operating procedures, real-time sharing of session content (e.g., through projecting content onto screens for multiple viewers to see), and identifiable point-people to support technology use appear to be tied to smoother delivery and more consistent use, in-turn reinforcing self-efficacy and social norms with respect to VR adoption. To mitigate ambivalence, both the literature and our study's participants endorse starting with social and low-risk goals (e.g., building reminiscence, relaxation, small-group activities) to demonstrate value and build momentum (53). They also recommended drawing on cultural-familial resources to select meaningful locations and contributions for VR sessions, factors that have been shown to boost engagement and ease burden (53). Our study advances existing findings by moving beyond descriptive accounts of ambivalence to VR adoption by linking them mechanistically through the DTPB to specific modifiable factors observed in real-world LTC, such as perceived ease-of-use, self-efficacy, social norms, and facilitating conditions. Addressing these factors and co-developing solutions with knowledge users allows VR to become a more feasible, low-burden adjunct intervention to pain-care by enabling the routine delivery of calming, individualized experiences that support relaxation, positive mood, and scaffolded resident involvement.

### 4.5 Strengths and limitations

This study has some limitations. The sample size was modest and situated within a specific LTC context with high neurocognitive disorder prevalence, limiting generalizability. Additionally, our sample involved convenience recruitment, potentially meaning that only LTC staff who showed the most interest in VR agreed to participate in both the training and the study. Furthermore, we were unable to collect information on the characteristics of LTC staff who declined to participate. This information would serve to enrichen the study's findings by building a more accurate profile of who potentially uses VR in LTC, vs. who is actually willing to share about their VR use. However, given the nuanced findings in the responses obtained, it appears that the impact of this sampling technique was relatively minor. It is also possible that because non-participating staff may have avoided the study due to negative experiences, our sample may not capture the full range of views. Including them might have yielded more critical perspectives. Another limitation is that we did not include perspectives from physicians, nurses, or nursing assistants who are typically involved in pain management in LTC. While our focus on frontline staff tasked with delivering VR programs to residents was intentional, future research should incorporate input from diverse clinical care perspectives to better understand how VR might complement pain-care strategies within interdisciplinary teams.

Questionnaire items were self-report and cross-sectional; some contained “unsure” responses, which are consistent with early adoption and variable training recency, but lead to reduced clarity regarding LTC staff's experiences. To address depth limitations inherent to survey-generated data, we complemented the dataset with qualitative analyses and a structured mixed-methods integration meeting, which involved research team members reviewing quantitative and qualitative findings together to confirm, expand, or identify divergences. Additionally, two separate two-hour online focus groups were conducted in April 2025 with ten LTC staff participants (five per group) who had completed the mixed-methods questionnaire analyzed in this study. These focus groups aimed to further test the robustness and transferability of themes and their findings will be compiled for future dissemination to healthcare, industry, and academic audiences. Finally, this study assessed a specific time-period of VR training and use. It does not account for developments in instruction and technology that have since been incorporated and which might have impacted LTC staff's perspectives on VR adoption.

However, this research is not without strengths. Our use of a convergent, mixed-methods design anchored in a theory-informed framework, enabled a more comprehensive interpretation of staff VR adoption alongside integration through the joint display of quantitative and qualitative findings. Second, it was conducted in a real-world LTC setting, enhancing ecological validity for later-life pain care. Third, we incorporated knowledge-user involvement throughout analysis and integration, which supported improved clarity in data collection materials and rapid, context-sensitive implementation actions (e.g., training updates, improvements to standard operating procedures). Fourth, our partnership model involving our neutral academic research team enabled us to link staff realities with organizational constraints and developer capacity to accelerate adoption. Fifth, VR has a favorable safety profile. Immersive sessions carry almost no serious side effects, making them a relatively low-risk intervention for LTC care.

An additional key strength of our scientific approach lies in the way we addressed challenges in collecting questionnaires. Based on recommendations from managers, we conducted in-person visits to meet staff, listen to their needs, provide training, and demonstrate how VR could be used with older adults. This strategy led us to visit 30 care facilities, which facilitated recruitment and encouraged participants to complete the questionnaires. These visits also fostered trust, helped us better understand potential challenges, and enabled the recruitment of additional participants for discussion groups, which will form the basis of future analyses and publications.

### 4.6 Future directions

Beyond these immediate results, our approach, grounded in direct engagement with LTC facilities through on-site visits, staff training, and collaborative implementation planning, sets the stage for a dynamic and iterative implementation process. The data collected in this study represent a first snapshot that can later be compared to new datasets, in order to evaluate whether modifications and adaptations are having a sustained impact. This continuous cycle of data collection and comparison will allow us to assess how implementation evolves over time and to identify further opportunities for improvement. In future phases, our laboratory aims to extend this comparative approach to include other facilities and care contexts, examining what works, what does not, and how differences across environments shape outcomes. These analyses will also benefit from the company's own datasets collected in parallel, enabling a more comprehensive understanding of barriers and facilitators and fostering a truly collaborative, evolving model of VR integration in care for older adults in LTC.

In line with our iterative approach, preliminary findings from this study were shared with the CISSS-CA and *Super Splendide* in April 2025. During a similar timeframe, the Toujours Dimanche application was iteratively refined based on feedback gathered by the company since 2022. These refinements included broadening the content catalogue from 64 to over 700 videos, with capacity for personal/family content and site-specific uploads, and implementing technical updates to enhance connectivity and streamline the user interface. Our team's role has been focused on informing staff about these updates and showing them how to access additional free applications (e.g., MetaTV, YouTube VR, Theater Elsewhere) to broaden the range of personalized content available for residents. A group-mode option for VR activities using HDMI cables and tablets has also been taught to staff to support shared viewing with residents, familiarization with different content, and gentle participant activation. For pain care, these adaptations are linked to mechanisms such as positive affect, distraction, presence, relaxation, and graded engagement, that are likely to support comfort and safe movement (86, 87).

Simultaneously, *Super Splendide* has been updating its training and supports based on feedback collected from staff trained within the CISSS-CA as well as from other training experiences in the multiple establishments where their technology has been deployed. In this context, the developer has been pursuing an iterative improvement process, releasing updated versions of the application and refining adoption supports in response to observed barriers and facilitators. Recent changes include an asynchronous, interactive online training (3–4 h long, delivered in 10–15-minute micro capsules) available on demand to accommodate busy schedules and staff turnover, followed by a comprehensive webinar to consolidate learning. The adoption process has also been revised to address learning needs, practice time, logistics, and change-management requirements, with additional planning support for managers. Following these modifications, an updated training program was reintroduced in summer 2025 and has continued into fall 2025. In parallel, our research team contributed to these efforts by accompanying LTC staff during VR implementation, identifying teams interested in research collaborations, and prioritizing direct, hands-on presence.

This preparatory work has thus laid the foundation for two funded projects to be implemented in the coming months: One project focuses on mindfulness to reduce chronic pain through Version 4 of the Toujours Dimanche app, while the other promotes physical activity using gradual exposure as part of the “Move Without Fear or Pain” program, supported by the VitaMove application developed by our team. Without this prior identification of barriers and facilitators, pilot projects would likely have encountered major feasibility issues.

## 5 Conclusion

Findings from our mixed-methods, cross-sectional study indicate that immersive VR has potential for successful implementation in later-life care when compatibility, ease-of-use, facilitating conditions, and social support align. LTC staff generally regarded immersive VR as beneficial, yet routine use depended on practical enablers like close alignment between resident needs and content, straightforward set-up and troubleshooting, protected time and appropriate space, and visible support from peers and leadership. Participants also pointed to the benefits of delivering sessions with personalized content and calm environments to enhance relaxation, positive mood, and consistency of use. While the findings reflect a specific setting and modest sample, they provide a clear, actionable basis for integrating VR into everyday LTC care rather than one-off activities. By articulating the specific thresholds and actions that enable routine delivery, and by operationalizing them with our partners, this work advances the field from promise toward practice, offering a foundation for future research on VR integration in LTC settings.

## Data Availability

The raw data supporting the conclusions of this article will be made available by the authors, without undue reservation.
