# Supplementary material for: Barriers and facilitators to implementing immersive virtual reality in long-term care settings: an interdisciplinary partnership study exploring staff perspectives
Source: Front Pain Res (Lausanne). 2026 Jan 29;7:1734386. doi: 10.3389/fpain.2026.1734386 (PMC12894301; doi:10.3389/fpain.2026.1734386)
Supplement: Supplementary file 1 [file Datasheet1.pdf]

Questions sociodémographiques

Veuillez prendre quelques instants pour répondre aux questions suivantes :

Âge :

____

Quel est le sexe qui vous a été attribué à la naissance, c'est-à-dire sur votre acte de naissance original ?

- ◯ Féminin
- ◯ Masculin
- ◯ Préfère ne pas répondre

Laquelle décrit le mieux votre identité de genre actuelle ? (C'est-à-dire, votre perception de vous-même) :

- ◯ Femme
- ◯ Homme
- ◯ Autre (svp spécifier) :
- ◯ Préfère ne pas répondre

Autre (svp préciser):

____

Quelle(s) catégorie(s) d'ethnicité vous décrit (décrivent) le mieux ? Sélectionnez toutes les options applicables :

- ☐ Asiatique de l'Est
- ☐ Asiatique du Sud-Est
- ☐ Asiatique du Sud
- ☐ Autochtone
- ☐ Blanc
- ☐ Latino-américain
- ☐ Moyen-oriental
- ☐ Noir
- ☐ Autre catégorie (svp spécifier)
- ☐ Ne sait pas
- ☐ Je préfère ne pas répondre

Ethnicité - Autre catégorie (svp spécifier) :

____

Langue maternelle :

____

Parlez-vous d'autres langues ?

- ◯ Oui
- ◯ Non

Si oui, svp spécifier :

____

Depuis combien d'années travaillez-vous dans un centre d'hébergement de soins de longue durée (CHSLD), maison des aînés (MDA), ou maison des aînés et alternatives (MDAA) ?

____

Quel est votre rôle au sein du CHSLD / MDA / MDAA ?

____
((p. ex., intervenant de loisir, physiothérapeute, infirmière))
