# Supplementary material for: Barriers and facilitators to implementing immersive virtual reality in long-term care settings: an interdisciplinary partnership study exploring staff perspectives
Source: Front Pain Res (Lausanne). 2026 Jan 29;7:1734386. doi: 10.3389/fpain.2026.1734386 (PMC12894301; doi:10.3389/fpain.2026.1734386)
Supplement: Supplementary file 2 [file Datasheet2.pdf]

Questionnaire

** no specific Social Norms q included, but did include peer influence and supervisor influence q

Le but de ce questionnaire est de mieux comprendre votre perception de l'utilisation de la réalité virtuelle afin d'améliorer l'utilisation de cet outil pour votre pratique avec votre clientèle.

À quelle fréquence avez-vous utilisé la réalité virtuelle?

Nombre moyen de fois par mois : __________________

En moyenne, vous utilisez la réalité virtuelle combien de temps par séance avec une personne résidente ? __________________

Combien d'heures avez-vous utilisé la réalité virtuelle au cours des 6 derniers mois ? Svp, répondez en précisant le temps total que vous avez consacré en séances individuelles et/ou de groupe : __________________

Combien de personnes résidentes voyez-vous par séance (en moyenne) ? __________________

Veuillez indiquer votre opinion concernant les énoncés suivants :

Utiliser la réalité virtuelle avec les personnes résidentes est une bonne idée.	◯ Oui	◯ Non	◯ Ne sais pas
La réalité virtuelle apporte quelque chose de plus que ce que mon approche conventionnelle peut offrir aux personnes résidentes.	◯ Oui	◯ Non	◯ Ne sais pas
Je trouve que la réalité virtuelle est facile à utiliser.	◯ Oui	◯ Non	◯ Ne sais pas
Les personnes résidentes démontrent que je devrais inclure la réalité virtuelle dans leurs séances d'activités.	◯ Oui	◯ Non	◯ Ne sais pas
Mes collègues me proposent d'utiliser la réalité virtuelle avec les personnes résidentes.	◯ Oui	◯ Non	◯ Ne sais pas
Mon superviseur me propose d'utiliser la réalité virtuelle avec les personnes résidentes.	◯ Oui	◯ Non	◯ Ne sais pas
J'ai assez de temps pour utiliser la réalité virtuelle dans mon horaire.	◯ Oui	◯ Non	◯ Ne sais pas
Je suis intéressé par l'utilisation de la réalité virtuelle avec les personnes résidentes.	◯ Oui	◯ Non	◯ Ne sais pas

Les personnes résidentes n'ayant jamais essayé la réalité virtuelle semblent motivées à utiliser cette technologie.	◯ Oui ◯ Non ◯ Ne sais pas
Les personnes résidentes ayant essayé la réalité virtuelle semblent motivées à réutiliser cette technologie.	◯ Oui<br>◯ Non<br>◯ Ne sais pas
J'ai le soutien dont j'ai besoin de la part de la direction pour utiliser la réalité virtuelle.	◯ Oui ◯ Non ◯ Ne sais pas
J'ai l'espace suffisant pour utiliser la réalité virtuelle.	◯ Oui ◯ Non ◯ Ne sais pas
J'ai suffisamment de temps durant ma journée de travail pour apprendre à utiliser la réalité virtuelle.	◯ Oui ◯ Non ◯ Ne sais pas
J'ai accès à suffisamment d'opportunités éducatives sur l'utilisation de la réalité virtuelle.	◯ Oui ◯ Non ◯ Ne sais pas
La réalité virtuelle cible efficacement les besoins des personnes résidentes.	◯ Oui ◯ Non ◯ Ne sais pas
J'ai l'intention d'utiliser la réalité virtuelle aussi souvent que nécessaire.	◯ Oui ◯ Non ◯ Ne sais pas
Je vais recommander aux autres intervenant(e)s d'utiliser la réalité virtuelle dans leur pratique.	◯ Oui ◯ Non ◯ Ne sais pas
J'ai eu suffisamment de formation avec Super Sublime pour apprendre à bien utiliser la réalité virtuelle.	◯ Oui ◯ Non ◯ Ne sais pas

À l'aide de l'échelle d'évaluation ci-dessous, veuillez évaluer votre confiance dans chacun des aspects suivants avec votre utilisation de la réalité virtuelle :

Mise en place de l'équipement de réalité virtuelle	◯ Très inconfortable<br>◯ Inconfortable<br>◯ Neutre<br>◯ Confortable<br>◯ Très confortable
Sélectionner les résidents appropriés pour participer à une séance de réalité virtuelle	◯ Très inconfortable ◯ Inconfortable<br>◯ Neutre ◯ Confortable<br>◯ Très confortable
Évaluer le niveau de participation et d'engagement des résidents	◯ Très inconfortable<br>◯ Inconfortable<br>◯ Neutre<br>◯ Confortable<br>◯ Très confortable

Gestion des problèmes techniques	◯ Très inconfortable ◯ Inconfortable ◯ Neutre ◯ Confortable ◯ Très confortable
Veuillez évaluer votre niveau de confiance dans l'utilisation de la réalité virtuelle avec vos résidents de 0% (pas du tout confiant) à 100% (extrêmement confiant) :	0% 50% 100%<br>*(Place a mark on the scale above)*
Pourquoi avez-vous sélectionner ce pourcentage?	__________________________________
Commentaires et suggestions afin d'améliorer votre niveau de confiance dans l'utilisation de la réalité virtuelle :	__________________________________
Avez-vous fait la formation en présentiel d'environ 7h avec Super Sublime pour l'utilisation de la réalité virtuelle avec les personnes résidentes ?	◯ Oui ◯ Non ◯ Ne sais pas
Veuillez indiquer la/les période/s pendant laquelle/lesquelles vous avez reçu votre formation avec Super Sublime pour l'utilisation de la réalité virtuelle avec les personnes résidentes :	☐ Printemps 2022 ☐ Été 2022 ☐ Automne 2022 ☐ Hiver 2022 ☐ Automne 2023 ☐ Ne se souvient pas
Avez-vous participé au relance webinaire de formation pour l'utilisation de la réalité virtuelle en printemps 2023?	◯ Oui ◯ Non ◯ Ne sais pas
Avez-vous fait la formation disponible en ligne (les capsules vidéos) grâce à la communauté de pratique de Super Sublime ?	◯ Oui ◯ Non ◯ Ne sais pas
Avez-vous déjà utilisé le support technique de Super Sublime ?	◯ Oui ◯ Non ◯ Ne sais pas
Veuillez évaluer la qualité de la formation que vous avez reçue avec Super Sublime pour l'utilisation de la réalité virtuelle avec les personnes résidentes de 0% (de très mauvaise qualité) à 100% (d'une excellente qualité) :	0% 50% 100%<br>*(Place a mark on the scale above)*
Pourquoi avez-vous sélectionner ce pourcentage?	__________________________________
Qu'avez-vous appris durant votre formation avec Super Sublime?	__________________________________

Quelle a été la durée de votre formation avec Super Sublime?	__________________
Est-ce que la formation en réalité virtuelle offerte par Super Sublime pourrait être améliorée?	◯ Oui ◯ Non
Si oui, quels éléments devraient être modifiés ou ajoutés au programme de formation pour l'utilisation de la réalité virtuelle?	__________________
Est-ce que les problèmes de vision étaient un enjeu important pour l'utilisation de la réalité virtuelle avec les résidents?	◯ Oui ◯ Non
Pourquoi? :	__________________
Est-ce que l'équipement semblait confortable pour vos résidents?	◯ Oui ◯ Non
Pourquoi? :	__________________
Avez-vous perçu des effets positifs de la réalité virtuelle auprès des personnes résidentes?	◯ Oui ◯ Non
Si oui, quel est l'effet perçu :	__________________
Avez-vous perçu des effets négatifs de la réalité virtuelle auprès des personnes résidentes ?	◯ Oui ◯ Non
Si oui, quel est l'effet perçu :	__________________
Quelles sont les barrières à l'utilisation de la réalité virtuelle (technologiques, humaine, temps, institutionnel/organisationnel, etc.)?	__________________
Comment pourrait-on améliorer l'expérience de la réalité virtuelle?	
Pour les personnes résidentes :	__________________
Pour les personnes intervenantes :	__________________

Pourquoi utilisez-vous la réalité virtuelle (traitement difficile, anxiété, activités, etc.)?	__________________________________
Est-ce que la réalité virtuelle semble adaptée à cette clientèle (en général, spécifique)?	◯ Oui ◯ Non
Pourquoi? :	__________________________________
Quelle utilisation de la réalité virtuelle serait pertinente avec cette clientèle?	__________________________________
Aviez-vous des idées préconçues sur la réalité virtuelle?	◯ Oui ◯ Non
Si oui, lesquels :	__________________________________
Avez-vous trouvé que ces idées préconçues sur la réalité virtuelle sont vraies?	◯ Oui ◯ Non ◯ Non applicable
Pourquoi?	__________________________________
Est-ce que votre perception de l'utilisation de la réalité virtuelle a changée après vos premières utilisations?	◯ Oui ◯ Non
Si oui, en quoi? :	__________________________________
Est-ce que la réalité virtuelle est utile?	◯ Oui ◯ Non
Pourquoi?	__________________________________
Pensez-vous maintenir l'utilisation de la réalité virtuelle à l'avenir?	◯ Oui ◯ Non
Pourquoi?	__________________________________
Merci d'ajouter ici tous commentaires supplémentaires par rapport à votre utilisation de réalité virtuelle que vous aimeriez partager à notre équipe de recherche :	__________________________________
